# Radiomics-based interpretable machine learning model from multiphasic CT imaging for predicting pathological grade in upper tract urothelial carcinoma: a multicenter study

**DOI:** 10.3389/fonc.2026.1844559

**Published:** 2026-06-23

**Authors:** Zhanpeng Yuan, Yuhua Mei, Xiang Peng, Zongjie Wei, Yingjie Xv, Bangxin Xiao, Mingzhao Xiao

**Affiliations:** Department of Urology, The First Affiliated Hospital of Chongqing Medical University, Chongqing, China

**Keywords:** CT urography, machine learning, pathological grade, radiomics, upper tract urothelial carcinoma

## Abstract

**Objective:**

This study aims to develop a non-invasive machine learning (ML) model based on radiomic features extracted from computed tomography urography (CTU) images to predict the pathological grade of upper tract urothelial carcinoma (UTUC), and aims to assist in preoperative diagnosis.

**Methods:**

A retrospective multicenter study was conducted with 338 patients who underwent radical nephroureterectomy (RNU) and preoperative CTU between June 2015 and June 2024. Radiomic features were extracted from non-contrast, arterial and venous CTU phases using the PyRadiomics. A total of 3,864 features were initially extracted, with feature selection performed using intraclass correlation coefficient, Spearman correlation, t-tests and LASSO regression. Six machine learning models (XGBoost, ExtraTrees, RandomForest, Support Vector Machine (SVM), Multi-Layer Perceptron (MLP), Light Gradient Boosting Machine (LGBM)) were employed to construct predictive models. Model performance was evaluated using receiver operating characteristic (ROC) curves, AUC, sensitivity, specificity and other metrics. SHAP method was used for model interpretability.

**Results:**

In the training set, LGBM demonstrated the highest discriminative performance, with an AUC of 0.945, and showed balanced sensitivity (84.5%) and specificity (91.2%). In the test set, LGBM maintained robust generalizability with an AUC of 0.829 (sensitivity: 71.8%, specificity: 77.4%), and outperformed other models, including SVM and MLP. SHAP analysis revealed that the most influential features for prediction were related to the venous and arterial CTU phases, enhancing the model’s interpretability.

**Conclusion:**

The developed radiomics-based ML model demonstrates excellent potential in predicting pathological grade for UTUC preoperatively. It provides a non-invasive, clinically interpretable tool for improving diagnostic accuracy and aiding in personalized treatment planning.

## 1 Introduction

Upper tract urothelial carcinoma (UTUC), a malignancy originating from the renal pelvis and ureter epithelium, accounts for only 5%-10% of all urothelial cancers (1). Despite its relatively low incidence, UTUC has attracted considerable attention due to its highly invasive biological behavior and the tendency for rapid progression (1, 2). Several risk factors contribute to the development and prognosis of UTUC, including environmental exposures like smoking, as well as patient demographics such as advanced age, which has been shown to significantly impact survival outcomes (3). Radical nephroureterectomy (RNU) remains the standard treatment for UTUC; however, a considerable number of cases experience local recurrence or distant metastasis post-surgery. This may be linked to insufficient preoperative assessment of tumor heterogeneity (1, 4, 5). It is noteworthy that in the management of urothelial carcinoma, pathological grade plays a crucial role in stratifying tumors into different prognostic groups, which provides essential prognostic information for treatment decisions and optimizes patient management. The European Association of Urology (EAU) guidelines identify pathological grade as one of the primary prognostic factors (1, 6, 7). Consequently, establishing an accurate preoperative grade prediction system has become a key challenge in achieving personalized treatment.

Currently, CT urography (CTU), with its unique advantages in tumor anatomical localization and three-dimensional reconstruction, has been widely incorporated into the routine preoperative evaluation of UTUC (8, 9). However, traditional image interpretation heavily relies on the subjective experience of clinicians, and predicting pathological grade based on CTU images remains challenging. In this context, radiomics, which integrates imaging big data analysis with artificial intelligence, has demonstrated significant potential. By deeply extracting quantitative imaging features, such as texture, shape, and gray level distributions, radiomics can reveal the relationship between imaging features and prognosis, offering a novel, non-invasive pathway for precision diagnosis and treatment (10, 11). Several studies have already shown that radiomics can effectively distinguish pathological grades (12, 13).

The application of radiomics has made significant strides in fields such as lung cancer, breast cancer, and prostate cancer, with numerous studies demonstrating its capability in improving diagnostic accuracy and prognostic prediction (14–16). Furthermore, our previous research has underscored the potential role of radiomics in assessing postoperative survival in bladder cancer and clear cell renal cell carcinoma (ccRCC), as well as in identifying malignant risks in cystic kidney diseases (17–20). Despite these advances, the application of radiomics in UTUC remains in its early stages, with limited studies exploring its full potential in this area.

This study aims to extract radiomic features from CTU images and develop a non-invasive machine learning (ML) model based on radiomics to predict the pathological grade. This model is intended to assist in clinical disease diagnosis, clarify pathological grade, and provide tool support for preoperative treatment optimization and prognostic stratification. Furthermore, the study will explore the interpretability of the radiomics-based machine learning model using SHAP analysis. Moreover, to our knowledge, this is the first multicenter study based on CT scans aimed at exploring the potential of radiomics in predicting pathological grade in UTUC.

## 2 Methods

### 2.1 Patients

**Figure 1:**
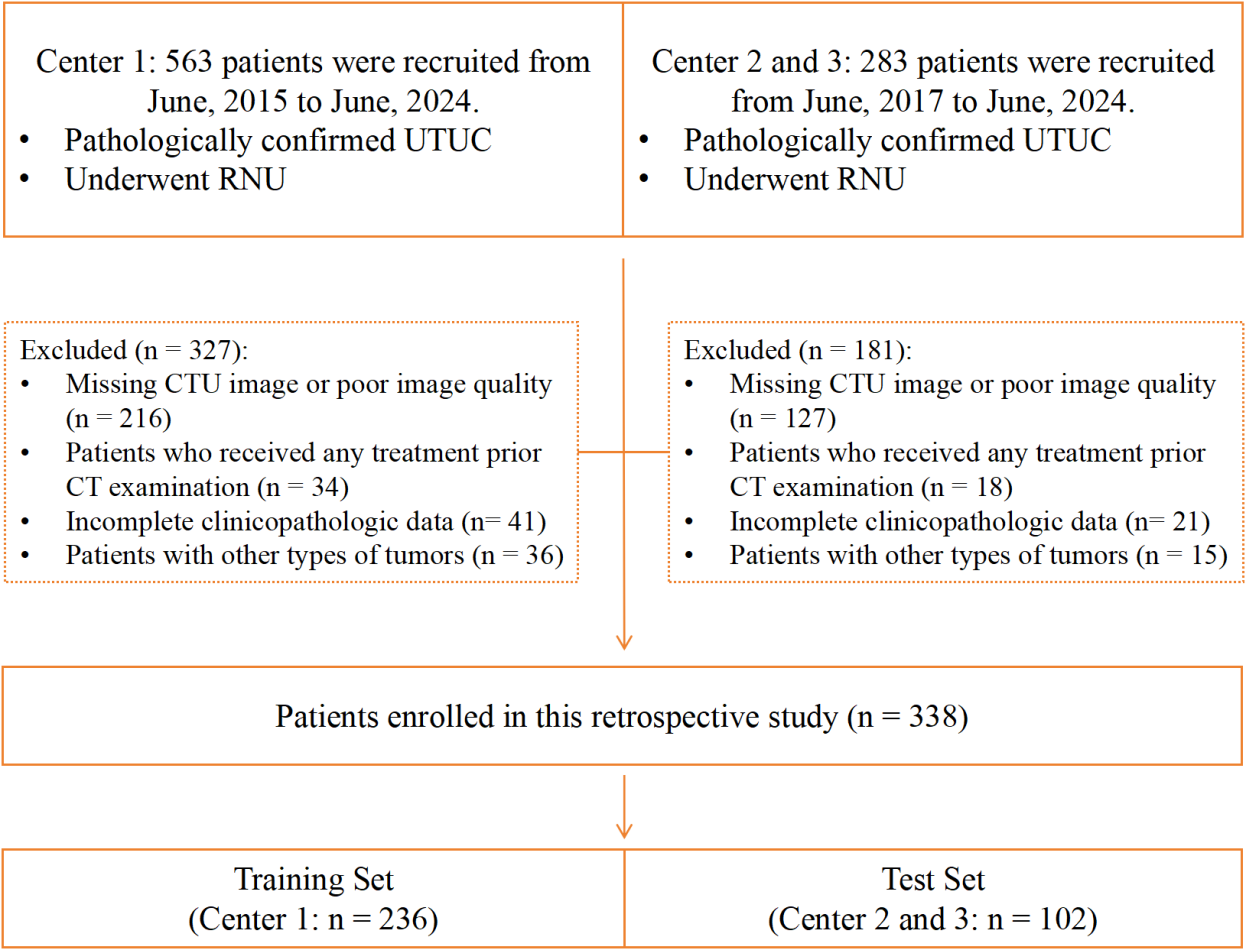
Flowchart of the patient inclusion process.

This retrospective multicenter study enrolled patients with histologically confirmed upper tract urothelial carcinoma (UTUC) who underwent radical nephroureterectomy (RNU) and preoperative computed tomography urography (CTU) from three independent hospitals. The inclusion criteria were as follows: 1) Postoperative pathological diagnosis of UTUC; 2) Underwent RNU; 3) Underwent CTU within a month before surgery without any intervening treatment. The exclusion criteria were as follows: 1) Postoperative pathological diagnosis not consistent with UTUC; 2) Loss or poor quality of CTU images; 3) Any treatment prior to CTU examination; 4) Incomplete clinical or pathological data; 5) Presence of other types of tumors. Ultimately, a total of 236 patients from June 2015 to June 2024 were included in the training set (Center 1) for model development, and 102 patients from June 2017 to June 2024 (Center 2 and 3) were included in the testing set. The patient enrollment and exclusion process is illustrated in **Figure 1**. This study was approved by the institutional ethics committee, and the requirement for informed consent was waived. Additionally, the following clinical and pathological data were collected from the electronic pathology system for each patient: age, gender, body mass index (BMI), smoking status, hydronephrosis status, laterality, tumor location, tumor size, lymphovascular invasion (LVI), T-stage, and margin status, among others.

### 2.2 UTUC pathological classification

The UTUC pathological grade was assessed using the WHO 2004/2016 classification system, which includes papillary urothelial neoplasm of low malignant potential, low-grade urothelial carcinoma, and high-grade urothelial carcinoma. The pathological grades were categorized into two major groups: LG (encompassing papillary urothelial neoplasm of low malignant potential and low-grade urothelial carcinoma) and HG (comprising high-grade urothelial carcinoma). Histopathological examination results were independently reviewed by two experienced pathologists with over 10 years of experience, who were blinded to the outcomes. Discordant results were discarded.

### 2.3 Imaging acquisition and ROI segmentation

All patients underwent CTU scans within 1 month before surgery. The specific details of the CT scanners and scanning parameters for each institution are provided in **Supplementary Table 1**. The CTU images were retrieved from the Picture Archiving and Communication Systems (PACS) and saved in the original Digital Imaging and Communications in Medicine (DICOM) format. In this study, we utilized the non-contrast, arterial, and venous phases of the CTU images for analysis.

The CTU images were segmented using ITK-SNAP software (version 3.6.0, http://www.itksnap.org). ITK-SNAP is a free, open-source, multi-platform application designed specifically for segmenting structures in 3D biomedical images. It offers a user-friendly interface and supports manual delineation of regions of interest (ROIs) in three orthogonal planes simultaneously. A urological radiologist with 5 years of experience (Reader A), who was blinded to the pathological and clinical information, manually segmented the ROIs of the tumors. For patients with multiple tumors, the lesion with the largest diameter was selected for ROI segmentation and subsequent feature extraction. To evaluate the inter- and intra-observer reproducibility of the radiomic feature extraction, 30 images were randomly selected for ROI segmentation by Reader A after a 2-week interval and by Reader B (with over 10 years of experience in diagnosing genitourinary diseases). Radiomic features with inter- and intra-class correlation coefficients (ICC) greater than 0.80 were considered to exhibit strong reliability and were retained for model construction. Additionally, the spatial overlap of the manually delineated ROIs between different observers and sessions was evaluated using the Dice Similarity Coefficient (DSC).

### 2.4 Radiomics features extraction and selection

All radiomics features were extracted using the open-source pyradiomics package in Python. The features were derived from the tumor ROIs in the non-contrast, arterial, and venous phases of CTU images. A total of seven categories of radiomics features were extracted: First Order features, Shape-based feature, Gray Level Co-occurrence Matrix (GLCM) features, Gray Level Run Length Matrix (GLRLM) features, Gray Level Size Zone Matrix (GLSZM) features, Gray Level Dependence Matrix (GLDM) features, and Neighboring Gray Tone Difference Matrix (NGTDM) features. The details of the radiomics feature extraction are shown in Supplementary Material: Appendix E1. Each radiomic feature was standardized using z-score normalization. Feature selection was performed in the training set according to the following steps: (1) ICC > 0.80; (2) Spearman correlation analysis; (3) Independent samples t-test (serving as a rigorous univariate pre-filtering step to eliminate pure noise features and reduce the extreme dimensionality of the multiphasic data); and (4) Least Absolute Shrinkage and Selection Operator (LASSO) regression algorithm with five-fold cross-validation.

### 2.5 ML model construction and evaluation

Six commonly used machine learning (ML) algorithms were employed to construct predictive models in the training set: XGBoost, ExtraTrees, RandomForest, Support Vector Machine (SVM), Multi-Layer Perceptron (MLP), and LightGBM (LGBM). These algorithms were chosen due to their robustness and ability to handle various types of data and model complexities. Specifically, XGBoost and LGBM are gradient boosting models that have proven effective for large datasets and imbalanced classes. ExtraTrees and RandomForest are ensemble tree-based methods known for their interpretability and high performance in various settings. SVM is a powerful method for classification with high-dimensional data, while MLP is a neural network-based model capable of capturing complex non-linear relationships.

**Figure 2:**
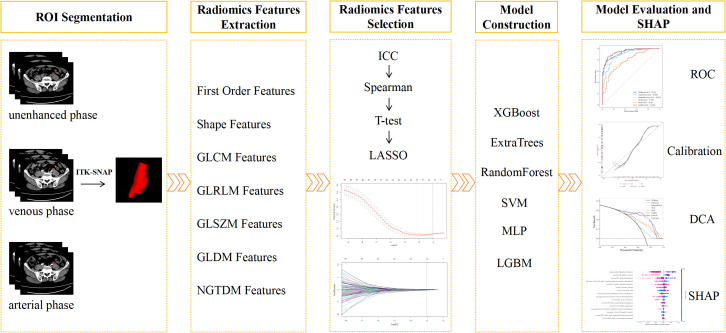
Schematic diagram of the overall workflow.

Receiver Operating Characteristic (ROC) curves and the Area Under the Curve (AUC) were used to evaluate the performance of the established ML models. The DeLong’s test was applied to compare the AUC between the models. Additionally, the accuracy (ACC), specificity (SPE), sensitivity (SEN), precision, and F1 score of each model were calculated within both the training and test sets. Model construction and performance evaluation were conducted using Python 3.12.0. To facilitate a more intuitive comparison of model performance, this study also utilized radar plots, Decision Curve Analysis (DCA), and calibration curves. The overall workflow of this study is depicted in **Figure 2**.

### 2.6 Model interpretability

To assess the interpretability of the ML models, SHAP (SHapley Additive exPlanations) values were used. SHAP is based on Shapley values from cooperative game theory, which allocate a contribution value to each feature based on its impact on the model’s prediction. The approach provides both local and global explanations of the model’s decision-making process.

The SHAP values were calculated using the SHAP package (version 2.0.0) to generate feature importance plot, summary plot, heatmap plot and force plots in Python. These plots were used to visualize the contribution of each feature and identify any significant relationships between features and the prediction outcomes.

### 2.7 Statistical analysis

All statistical analyses were performed using R software (version 4.1.2) and IBM SPSS (version 27.0.1). Continuous variables were expressed as means with standard deviations (SD), and categorical variables were presented as frequencies and percentages. The 95% confidence intervals (CI) for model performance metrics were computed using the bootstrapping method. Comparisons of clinical characteristics between the training and test sets were conducted using the chi-square test (or Fisher’s exact test) for categorical variables and the t-test (or Mann-Whitney U test) for continuous variables. All statistical tests were two-sided, and a p-value of less than 0.05 was considered statistically significant.

## 3 Results

### 3.1 Baseline characteristics

A total of 338 patients were included in this study, with 236 in the training set and 102 in the test set. The mean age of the overall population was 66.93 ± 9.97 years, with a male-to-female ratio of 199:139 (58.9% vs. 41.1%). The majority of patients had a BMI < 25 kg/m² (66.0%) and a history of non-smoking (63.3%), while 57.4% had hydronephrosis. Regarding tumor characteristics, 59.8% of patients had left-sided tumors, and 73.4% had tumors with a maximum diameter ≥ 2 cm. Most patients were staged as T2-T3 (66.9%), 10.1% had lymphovascular invasion (LVI), 7.7% had lymph node metastasis, and 70.7% are HG (**Table 1**). No statistically significant differences were observed between the training and test sets in baseline clinical characteristics (all p > 0.05), including demographic factors (age, sex, BMI), pathological features, and prognostic indicators. These results indicate that the baseline characteristics of the training and test sets were well-matched (p > 0.05), minimizing the potential for selection bias to affect model validation.

**Table 1:** Baseline characteristics of UTUC patients.

Characteristic	Total	Training set	Test set	p-value
	(n = 338)	(n = 236)	(n = 102)	
Age (years)	66.93±9.97	67.04±10.16	66.68±9.57	0.290
Gender				0.094
Male	199 (58.9%)	132 (55.9%)	67 (65.7%)	
Female	139 (41.1%)	104 (44.1%)	35 (34.3%)	
BMI (kg/m²)				0.860
≥25	115 (34.0%)	81 (34.3%)	34 (33.3%)	
<25	223 (66.0%)	155 (65.7%)	68 (66.7%)	
Smoker				0.260
Yes	124 (36.7%)	82 (34.7%)	42 (41.2%)	
No	214 (63.3%)	154 (65.3%)	60 (58.8%)	
Hydronephrosis				0.556
Yes	194 (57.4%)	133 (56.4%)	61 (59.8%)	
No	144 (42.6%)	103 (43.6%)	41 (40.2%)	
Laterality				0.329
Left	202 (59.8%)	137 (58.1%)	65 (63.7%)	
Right	136 (40.2%)	99 (41.9%)	37 (36.3%)	
Location				0.508
Pelvis	170 (50.3%)	117 (49.6%)	53 (52.0%)	
Ureter	151 (44.7%)	105 (44.5%)	46 (45.1%)	
Both	17 (5.0%)	14 (5.9%)	3 (2.9%)	
Size (cm)				0.563
≥2	248 (73.4%)	171 (72.5%)	77 (75.5%)	
<2	90 (26.6%)	65 (27.5%)	25 (24.5%)	
LVI				0.918
Yes	34 (10.1%)	24 (10.2%)	10 (9.8%)	
No	304 (89.9%)	212 (89.8%)	92 (90.2%)	
T-stage				0.544
Ta	30 (8.9%)	21 (8.9%)	9 (8.8%)	
Tis	14 (4.1%)	10 (4.2%)	4 (3.9%)	
T1	62 (18.3%)	47 (19.9%)	15 (14.7%)	
T2	105 (31.1%)	71 (30.1%)	34 (33.3%)	
T3	121 (35.8%)	81 (34.3%)	40 (39.2%)	
T4	6 (1.8%)	6 (2.5%)	0	
Margin				0.358
Positive	13 (3.8%)	11 (4.7%)	2 (2.0%)	
Negative	325 (96.2%)	225 (95.3%)	100 (98.0%)	
pN				0.945
N0/Nx	312 (92.3%)	218 (92.4%)	94 (92.2%)	
N+	26 (7.7%)	18 (7.6%)	8 (7.8%)	
Grade				0.770
High	239 (70.7%)	168 (71.2%)	71 (69.6%)	
Low	99 (29.3%)	68 (28.8%)	31 (30.4%)	

### 3.2 Feature selection

**Figure 3:**
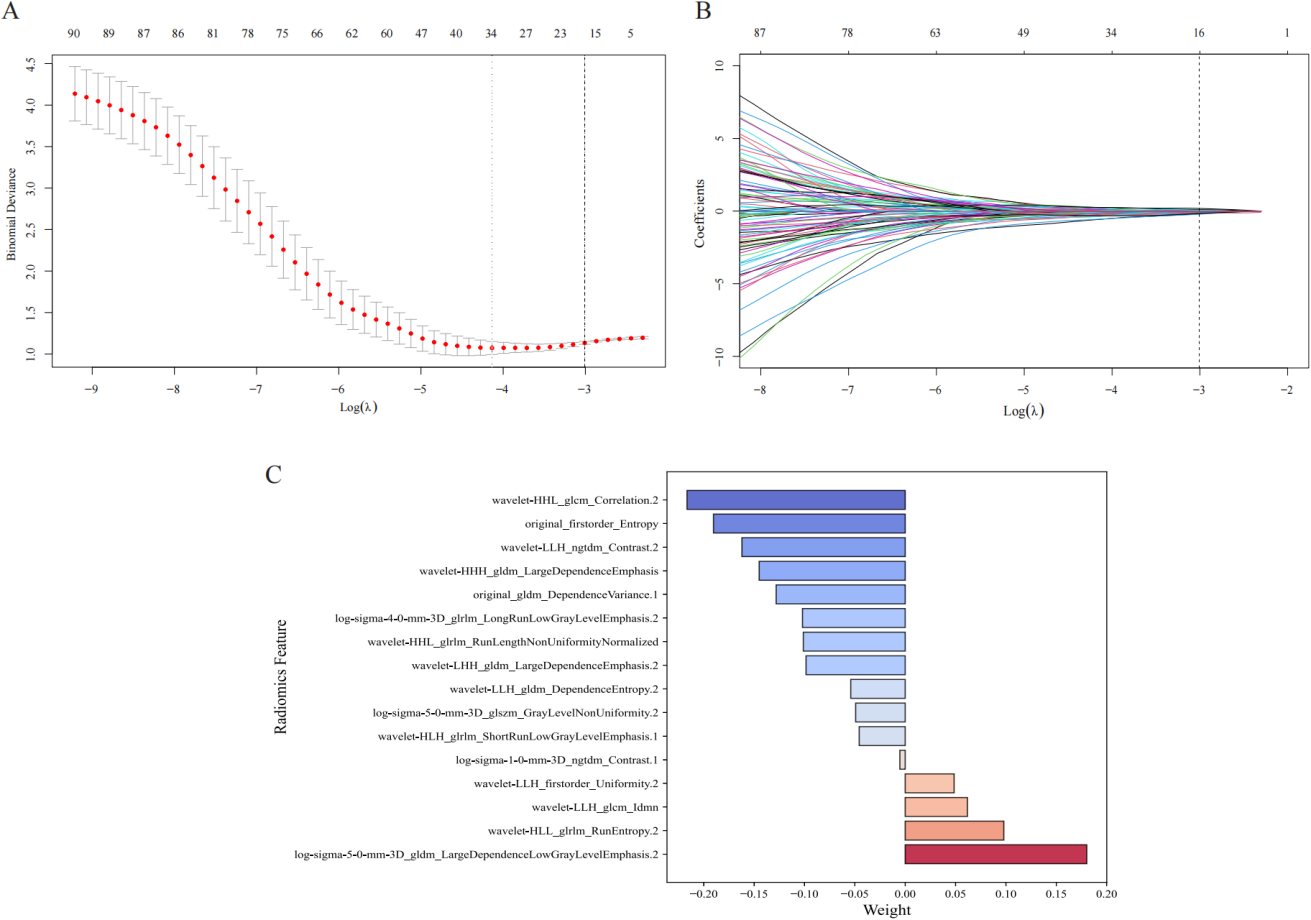
Radiomics feature selection process and corresponding weights. **(A)** LASSO coefficient profiles of the radiomic features across various tuning parameters (λ). The vertical dashed line indicates the optimal λ value (0.049) selected via five-fold cross-validation. **(B)** Tuning parameter (λ) selection in the LASSO model using five-fold cross-validation. The optimal features were selected at the minimum criteria. **(C)** The final weighting coefficients of the 16 selected features. Note: The suffixes in the feature names indicate the corresponding CTU phases: no suffix represents the non-contrast phase, ‘.1’ denotes the venous phase, and ‘.2’ denotes the arterial phase.

In this study, a total of 3,864 radiomics features were extracted from the non-contrast, arterial, and venous phases of CT urography (CTU) using the open-source PyRadiomics platform. To ensure feature reliability, we first applied intraclass correlation coefficient (ICC > 0.80) to exclude features with low inter-observer consistency, resulting in 3,306 reproducible features. Concurrently, the segmentation geometric consistency was highly robust, yielding a mean inter-observer DSC of 0.86 ± 0.05 and a mean intra-observer DSC of 0.89 ± 0.04. Subsequently, Spearman correlation analysis was conducted to remove highly correlated features, yielding 956 non-redundant features. A t-test (significance level, p < 0.05) was used to select 105 features significantly associated with clinical outcomes. Finally, a radiomics model was constructed using 16 predictive radiomics features identified through LASSO regression combined with five-fold cross-validation: 4 features from the non-contrast phase, 3 from the venous phase, and 9 from the arterial phase (**Figures 3A, B**). **Figure 3C** and **Supplementary Figure 1** present the feature weights and correlation heatmaps, respectively. In the feature nomenclature, the suffixes denote the specific CT phase from which the feature was extracted: no suffix indicates the non-contrast phase, ‘.1’ denotes the venous phase, and ‘.2’ denotes the arterial phase.

### 3.3 Model performance

**Figure 4:**
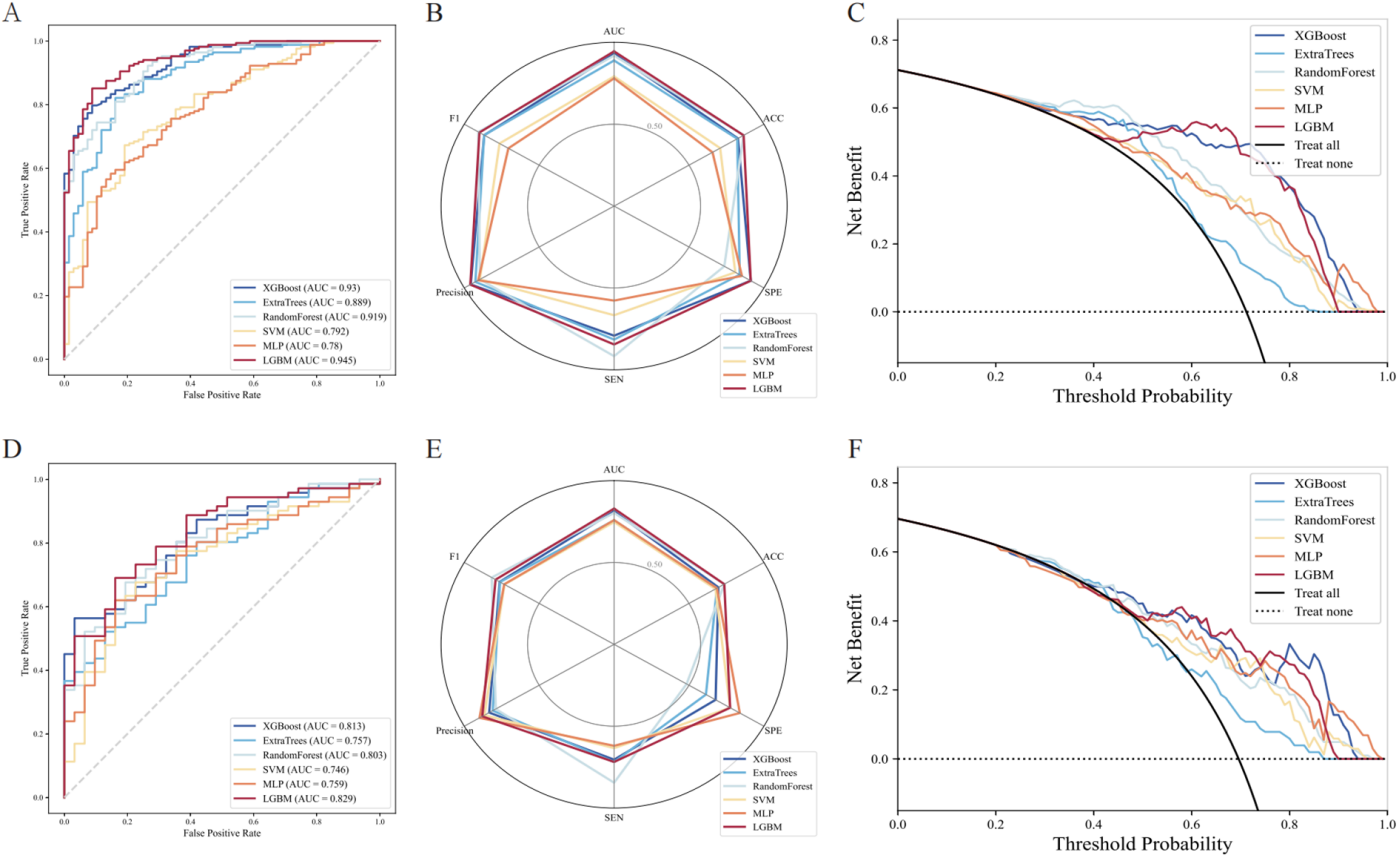
Comparison of ML model performance in predicting HG and LG. **(A–C)** The ROC curve, performance radar chart, and DCA of the ML models in the training set, respectively. **(D–F)** The ROC curve, performance radar chart, and DCA of the ML models in the test set, respectively. ML, machine learning; AUC, area under the curve; ROC, receiver operating characteristic; DCA, decision curve analysis.

The selected 16 radiomics features were input into six machine learning models. In the training set, Light Gradient Boosting Machine (LGBM) demonstrated the highest discriminative performance, achieving an AUC of 0.945 (95% CI: 0.915–0.971), significantly outperforming SVM (AUC = 0.792, p < 0.001) and MLP (AUC = 0.780, p < 0.001), while showing comparable accuracy to XGBoost (AUC = 0.930, p = 0.237) (**Table 2**, **Figures 4A, B**).

**Table 2:** Comparison of ML models’ performance on the training and test set.

Models		AUC	ACC	SPE	SEN	Precision	F1
Training set	XGBoost	0.930 (0.896-0.957)	0.826	0.912	0.792	0.957	0.866
	ExtraTrees	0.889 (0.841-0.934)	0.822	0.838	0.815	0.926	0.867
	RandomForest	0.919 (0.878-0.951)	0.864	0.735	0.917	0.895	0.906
	SVM	0.792 (0.722-0.848)	0.708	0.809	0.667	0.896	0.765
	MLP	0.780 (0.716-0.840)	0.657	0.853	0.577	0.907	0.705
	LGBM	0.945 (0.915-0.971)	0.864	0.912	0.845	0.959	0.899
Test set	XGBoost	0.813 (0.728-0.885)	0.696	0.677	0.704	0.833	0.763
	ExtraTrees	0.757 (0.657-0.847)	0.686	0.613	0.718	0.810	0.761
	RandomForest	0.803 (0.705-0.881)	0.735	0.484	0.845	0.789	0.816
	SVM	0.746 (0.648-0.846)	0.676	0.774	0.634	0.865	0.732
	MLP	0.759 (0.661-0.846)	0.686	0.839	0.620	0.898	0.733
	LGBM	0.829 (0.737-0.906)	0.735	0.774	0.718	0.879	0.791

AUC, Area Under the Curve; ACC, Accuracy; SPE, Specificity; SEN, Sensitivity.

In the test set, LGBM maintained robust generalizability, attaining the highest AUC of 0.829 (95% CI: 0.737–0.906), which was 3.4% superior to XGBoost (p = 0.537) and 10.9% higher than SVM (p = 0.040) (**Figures 4D, E**). Notably, LGBM demonstrated balanced sensitivity (71.8%) and specificity (77.4%) in the test set, with the smallest performance degradation from training to test, indicating effective mitigation of overfitting (**Table 2**). Furthermore, to evaluate the impact of scanner heterogeneity, a per-center performance breakdown for the external test set was conducted. The LGBM model demonstrated consistent predictive robustness across both Center 2 and Center 3 independently, maintaining stable AUCs and accuracy, as detailed in **Supplementary Table 3**.

Statistical comparisons using Delong’s test confirmed LGBM’s significant advantages over SVM (p = 0.016) and MLP (p = 0.028) in the test cohort (**Supplementary Table 2**). The optimal calibration curve and the highest clinical net benefit across threshold probabilities further supported its selection (**Figures 4C, F** and **Supplementary Figure 2**). Based on these results, LGBM was chosen as the final model due to its stable generalization performance and clinically interpretable risk stratification capability.

### 3.4 Model interpretability

**Figure 5:**
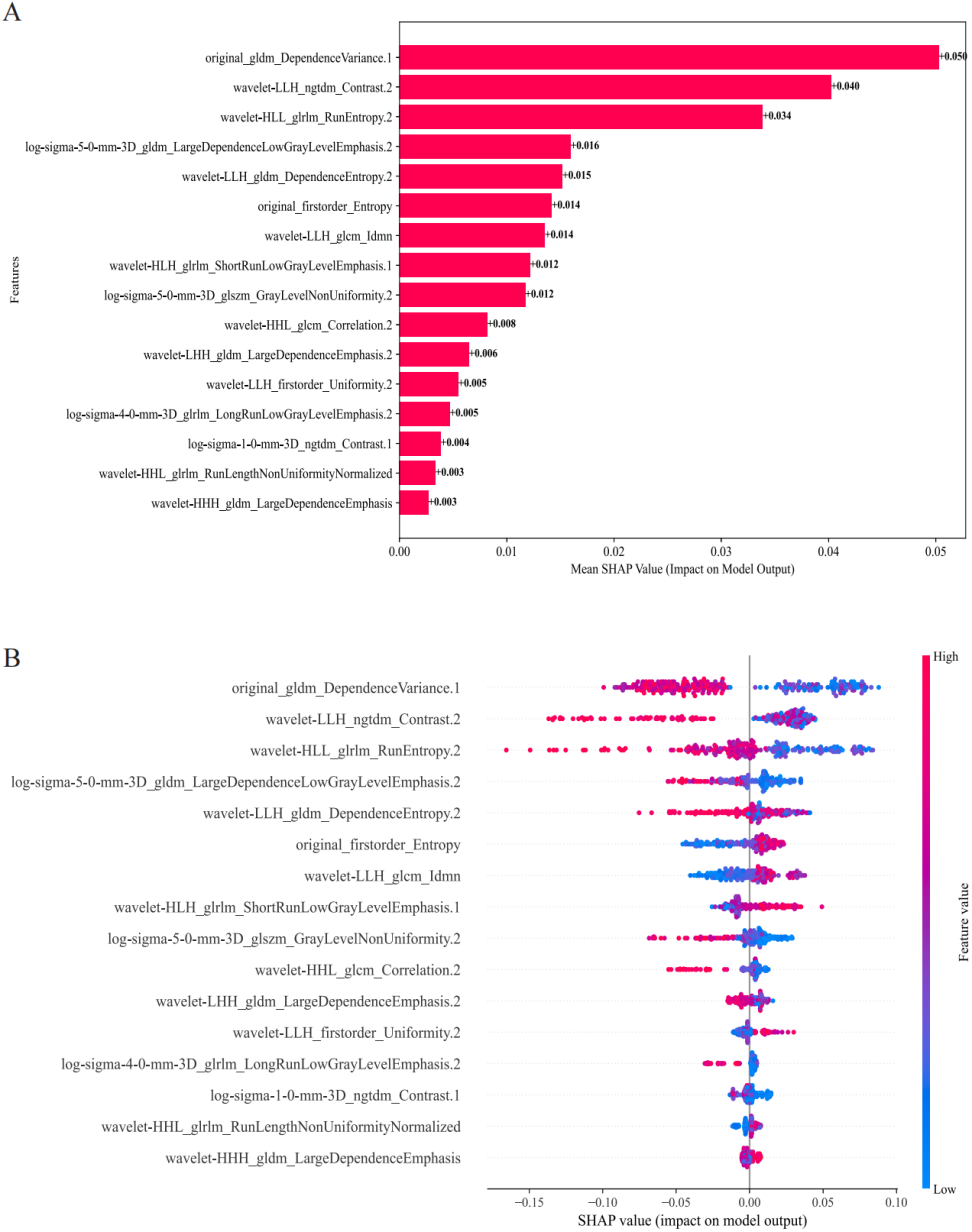
Evaluation of ML model radiomics feature interpretability using the SHAP method. **(A)** The SHAP bar plot displayed the global importance of each feature based on the mean SHAP values. **(B)** The SHAP summary plot showed the impact of individual radiomics features on the model’s predictions. Each dot represents a single patient, and the color gradient reflects the magnitude of the feature’s contribution to the model prediction, with red indicating a strong positive contribution and blue indicating a strong negative contribution. ML, machine learning; SHAP, Shapley additive explanation. The suffixes in the feature names indicate the corresponding CTU phases: no suffix represents the non-contrast phase, ‘.1’ denotes the venous phase, and ‘.2’ denotes the arterial phase.

**Figure 6:**
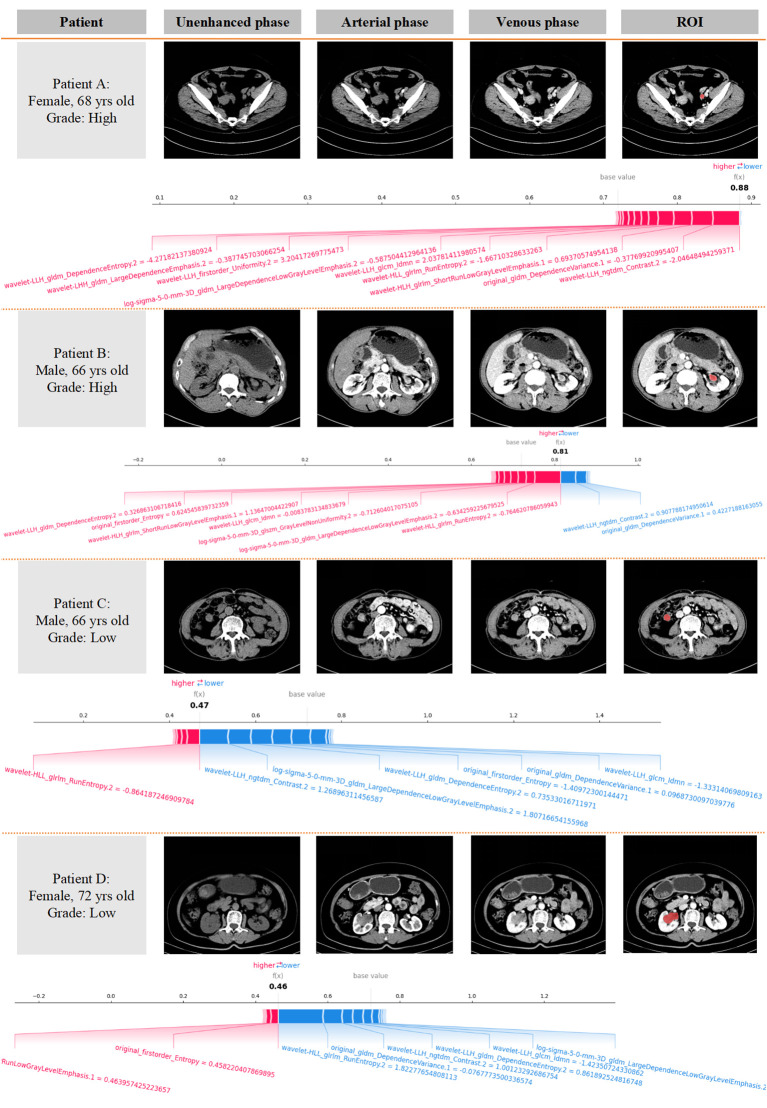
SHAP force plot visualization for representative cases. Four representative cases correctly predicted as HG [patients **(A, B)**] and LG [patients **(C, D)**] were individually visualized by SHAP method. SHAP, Shapley additive explanation.

Finally, the SHAP values for each imaging feature in the LGBM model were calculated. As shown in **Figure 5A**, the SHAP bar plot illustrates the global importance of each feature, with the top three features contributing the most to the model’s predictions: original_gldm_DependenceVariance from the venous phase, wavelet-LLH_ngtdm_Contrast from the arterial phase, and wavelet-HLL_glrlm_RunEntropy from the arterial phase. We also generated SHAP summary plot to further elucidate the impact of individual features on the model’s output (**Figure 5B**). Additionally, a SHAP heatmap was created to visualize feature importance and the relationships between features, enhancing the interpretability of the model (**Supplementary Figure 3**). Lastly, **Figure 6** presents four representative cases with correct predictions of pathological HG and LG outcomes, demonstrating the model’s clinical applicability.

## 4 Discussion

In this study, we developed and externally validated an interpretable machine learning (ML) model for predicting pathological grade in upper tract urothelial carcinoma (UTUC). The model was constructed based on radiomic features extracted from the CTU non-contrast phase, arterial phase and venous phase. In recent years, risk stratification based on pathological grade has played a key role in prognostic assessment and treatment decision-making for patients with UTUC (1, 21, 22). However, current pathological grade of UTUC primarily relies on postoperative histological examination, a method that is time-consuming and invasive (23). Therefore, the development of an efficient, non-invasive predictive tool is critically important. The model proposed in this study not only improves the accuracy of pathological grade prediction for UTUC but also provides a novel non-invasive approach for personalized treatment.

In recent years, the role of radiomics in early disease diagnosis and prognostic prediction has been extensively researched (24–26). For example, Peng et al. (27) used MRI imaging data to differentiate between HER2-Zero, -Low, and -Positive breast cancer patients, aiding in the selection of patients for novel or traditional HER2-targeted therapies. Maria Thor et al. (28) explored and compared published and new pre-treatment CT and PET radiomics, stratifying the progression risk of early-stage non-small cell lung cancer (NSCLC) patients undergoing stereotactic radiotherapy, with a C-index of 0.78. Similarly, the application of imaging techniques in UTUC has garnered increasing attention, and several studies have attempted to construct predictive models based on single-center data. For instance, Zheng et al. (29) incorporated CTU data from a single-center UTUC cohort to establish a machine learning model in predicting preoperative pathological grade. These studies have demonstrated the feasibility of CT-based radiomics in pathological grade. However, most of these studies lack external validation, which often leads to models overfitting to specific institutional imaging protocols, thereby limiting their model generalizability and stability. In this study, we integrated data from three independent hospitals, which inherently incorporates the real-world heterogeneity of different CT scanners and acquisition parameters. The robust performance of our model across these diverse external datasets more forcefully validates its generalization capability compared to single-center models, demonstrating its reliability for broader clinical application. This approach not only improved the reliability of the model but also provides valuable experience for future multi-center machine learning studies. Furthermore, comparing our proposed model with conventional preoperative modalities underscores its substantial clinical value. In routine clinical practice, urine cytology suffers from notoriously low sensitivity in detecting LG lesions, while traditional radiologist interpretation of CTU images is highly subjective and lacks standardized criteria for precise grading. Although ureteroscopic biopsy is widely utilized, it remains invasive, carries risks of tumor seeding or strictures, and is significantly hindered by sampling bias due to limited tumor fragments. Notably, a previous multi-institutional study demonstrated that up to 51% of patients with clinical LG tumors on biopsy were upgraded to pathological HG tumors at final surgical pathology (23). In contrast, our non-invasive LGBM model achieved a robust AUC of 0.829 in the independent external test set, offering a highly reliable, objective, and risk-free decision-support tool to complement pre-operative biopsy and mitigate such clinical diagnostic discordance.

Regarding the selection and optimization of machine learning models, we employed several classic algorithms, including XGBoost, RandomForest, Support Vector Machine (SVM), and LightGBM (LGBM). Ultimately, the LGBM algorithm exhibited the best performance, with AUCs of 0.945 and 0.829 in the training and test sets, respectively, significantly outperforming the other algorithms. This result indicates that LGBM is not only effective in handling high-dimensional data but also demonstrates strong stability and low risk of overfitting, even in small sample sizes. For example, He et al. (30) used LGBM to predict epidermal growth factor mutations in lung adenocarcinoma (AUC = 0.94). Additionally, LGBM models have shown good performance in multi-parametric imaging radiomics using conventional T1-weighted and susceptibility-weighted imaging for distinguishing between idiopathic Parkinson’s disease and multiple system atrophy (31), confirming the broad applicability of LGBM in medical image analysis. Our study also provides useful insights for future imaging radiomics research in other cancer types using LGBM. The moderate decline in AUC from the training set (0.945) to the external test set (0.829) is a well-recognized challenge in radiomics, primarily driven by multi-institutional imaging heterogeneity. As shown in **Supplementary Table 1**, variations in scanner manufacturers and tube current settings across different centers inherently introduce distinct image reconstruction algorithms and varying noise levels. These technical factors inevitably affect the consistency of radiomic feature expressions. Nevertheless, our proposed LGBM model successfully maintained a robust AUC above 0.81 in both independent external datasets (**Supplementary Table 3**), forcefully validating its generalizability and clinical reliability despite real-world scanning variations.

Furthermore, the interpretability of machine learning models is crucial for their widespread clinical adoption. In this study, we performed an interpretability analysis of the LGBM model using SHAP values, which revealed the impact of key imaging features on the model’s predictions. SHAP values, based on game theory, quantify each feature’s contribution to the model’s decision, thereby providing intuitive imaging explanations for clinicians (32, 33). Through SHAP visualization tools, we further enhanced the transparency of the model and increased its clinical acceptability. In our study, global feature importance analysis showed that the “original_gldm_DependenceVariance” in the venous phase, and “wavelet-LLH_ngtdm_Contrast” and “wavelet-HLL_glrlm_RunEntropy” in the arterial phase played crucial roles in prediction, providing reliable imaging biomarkers for pathological grade of UTUC. Notably, “NGTDM” features have been shown to be useful for constructing models predicting pathological grade in bladder cancer (34). Biologically, these top-weighted mathematical radiomic properties reflect the intrinsic pathophysiological and microenvironmental alterations associated with tumor differentiation (12, 13). Specifically, original_gldm_DependenceVariance in the venous phase measures the variance in gray-level dependencies, capturing the high degree of structural disorder, irregular cellular density, and heterogeneous necrosis typically found within aggressive, high-grade UTUC tissue. On the other hand, wavelet-LLH_ngtdm_Contrast in the arterial phase quantifies the local intensity difference and spatial texture contrast. During the arterial active perfusion phase, high-grade tumors frequently exhibit rapid, chaotic neo-angiogenesis and arteriovenous shunting, leading to highly uneven, patchy, and disorganized contrast enhancement (34, 35). By capturing these microscopic tissue alterations that are otherwise invisible to the naked eye, these radiomic features serve as non-invasive macroscopic surrogates for underlying tumor biology. Furthermore, to better understand how individual features influence the prediction outcomes, we visualized SHAP force plots for specific instances.

Although this study provides promising results, several limitations remain. First, although we used multi-center data, the sample size is still relatively small. Future studies with larger datasets, particularly those from diverse regions and populations, will be essential for enhancing the model’s generalizability and reliability. Second, this study relied solely on CTU imaging data and did not incorporate other imaging modalities, such as MRI. Third, all three participating clinical centers in this study are located within China, which inherently restricts the demographic profile to an Asian population and may limit the direct generalizability of our predictive model to Western cohorts. Further validation within global, trans-ethnic multi-center registries is warranted to refine the clinical utility of our framework. Lastly, manual delineation of regions of interest (ROIs) by clinicians may introduce potential biases and is labor-intensive. To mitigate this, future research should prioritize the development of semi-automated or AI-assisted segmentation frameworks. Given the unique anatomical complexity of the upper urinary tract—where tumors often exhibit infiltrative growth within narrow lumens—semi-automated tools could maintain high precision while significantly reducing manual effort. Furthermore, long-term improvements could involve training customized deep learning architectures (e.g., 3D U-Net) specifically optimized for the urinary collecting system. Therefore, future research should focus on expanding the dataset, integrating multiple imaging techniques, and optimizing the automation and standardization of AI models to further improve the accuracy, generalizability, and clinical utility of these models.

## 5 Conclusion

In conclusion, this study developed an accurate predictive model for pathological grade of UTUC by integrating multi-center CTU imaging data and ML algorithm. The model demonstrates high predictive accuracy, stability, and interpretability, providing strong support for personalized treatment decisions.

## Data Availability

The original contributions presented in the study are included in the article/**Supplementary Material**, further inquiries can be directed to the corresponding author/s.
