# Supplementary material for: Radiomics-based interpretable machine learning model from multiphasic CT imaging for predicting pathological grade in upper tract urothelial carcinoma: a multicenter study
Source: Front Oncol. 2026 Jun 23;16:1844559. doi: 10.3389/fonc.2026.1844559 (PMC13337430; doi:10.3389/fonc.2026.1844559)
Supplement: Supplementary file 1 [file DataSheet1.docx]

Supplementary Material

**Appendix E1: The details of radiomics feature extraction of this study**

In preparation for radiomics feature extraction, all images were resampled to symmetric voxels of 1×1×1 mm³ using spline interpolation. The CT-based feature extraction was carried out using the pyradiomics package in Python (version 3.12.0), following the protocols established by the Image Biomarker Standardization Initiative (IBSI).

Seven kinds of radiomics features were extracted in this study:

1. First-order features: describe the distribution of voxel intensities within the ROIs through commonly used and basic metrics.
2. Shape-based features: describe the shape and size of ROIs.
3. Gray Level Co-occurrence Matrix (GLCM) Features: describe how frequently a certain combination of pixel values (like brightness levels) occurs in two pixels that are a certain distance apart, along a specific angle.
4. Gray Level Size Zone Matrix (GLSZM) Features: quantifies gray level zones in an image.
5. Gray Level Run Length Matrix (GLRLM) Features: quantifies gray level runs, which are defined as the length in number of pixels, of consecutive pixels that have the same gray level value.
6. Neighbouring Gray Tone Difference Matrix (NGTDM) Features: quantifies the difference between a gray value and the average gray value of its neighbours within distance.
7. Gray Level Dependence Matrix (GLDM) Features: quantifies gray level dependencies in an image.

Detailed descriptions and mathematic formulas of each radiomics feature are shown on <https://pyradiomics.readthedocs.io/en/latest/features.html.>

# Supplementary Tables

**Table S1** The CT protocols of the three centers.

| Parameters | Center 1 | Center 2 | Center 3 |
| --- | --- | --- | --- |
| CT version | Discovery CT750 HD (GE Healthcare, USA) or SOMATOM Definition Flash (Siemens Healthcare, Germany) | SOMATOM Definition AS (Siemens Healthcare, Germany); Revolution CT (GE Healthcare, USA) | Aquilion One (Toshiba Medical Systems, Japan); LightSpeed VCT (GE Healthcare, USA) |
| CT tube voltage | 100-120 kV | 100-120 kV | 110-120 kV |
| CT tube current | 200-500 mA | 100-250 mA | 250-450 mA |
| Gantry rotation time | 0.50-0.60 s | 0.28-0.33s | 0.50s |
| Detector collimation (mm) | 0.625 mm | 0.6-0.625 mm | 0.625 mm |
| Image matrix | 512*512 | 512*512 | 512*512 |
| Slice thickness | 5-7 mm | 1-5 mm | 1-7 mm |

**Table S2** Comparisons of AUC bewteen ML models by Delong’s test in the training set and test set.

| Models | Training set | | | Test set | | |
| --- | --- | --- | --- | --- | --- | --- |
|  | AUC | *p* value^*^ | *p* value^**^ | AUC | *p* value^*^ | *p* value^**^ |
| XGBoost | 0.930 | 0.237 | - | 0.813 | 0.537 | - |
| ExtraTrees | 0.889 | 0.006 | 0.007 | 0.757 | 0.045 | 0.079 |
| RandomForest | 0.919 | 0.079 | 0.386 | 0.803 | 0.379 | 0.684 |
| SVM | 0.792 | <0.001 | <0.001 | 0.746 | 0.040 | 0.081 |
| MLP | 0.780 | <0.001 | <0.001 | 0.759 | 0.046 | 0.084 |
| LGBM | 0.945 | - | 0.237 | 0.829 | - | 0.537 |

^*^Delong’s test was employed to compare the AUC between the LGBM with XGBoost, ExtraTrees, RandomForest, SVM and MLP models, respectively.

^**^Delong’s test was employed to compare the AUC between the XGBoost with LGBM, ExtraTrees, RandomForest, SVM and MLP models, respectively.

**Table S3** The per-center performance breakdown of the LGBM model in the test set.

|  | **Center 2 (n = 64)** | **Center 3 (n = 38)** |
| --- | --- | --- |
| **AUC (95% CI)** | 0.819 (0.692-0.919) | 0.842 (0.696-0.954) |
| **ACC** | 0.734 | 0.737 |
| **SPE** | 0.706 | 0.857 |
| **SEN** | 0.745 | 0.667 |
| **Precision** | 0.875 | 0.889 |
| **F1** | 0.805 | 0.762 |

## Supplementary Figures


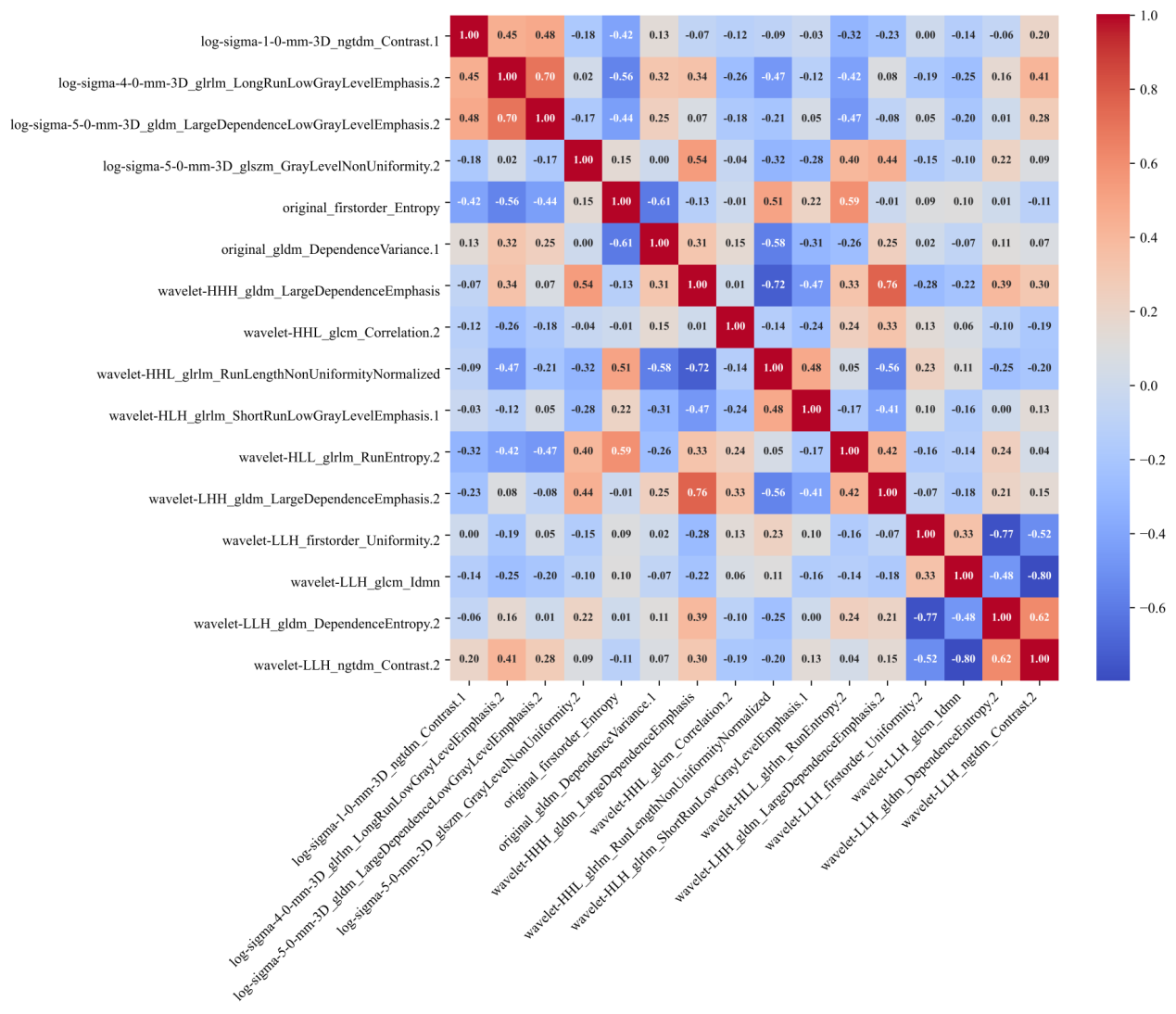


**Fig.S1** The heatmap showing the correlation between radiomics features selected by the LASSO regression algorithm. Note: The suffixes in the feature names indicate the corresponding CTU phases: no suffix represents the non-contrast phase, ‘.1’ denotes the venous phase, and ‘.2’ denotes the arterial phase.

A B


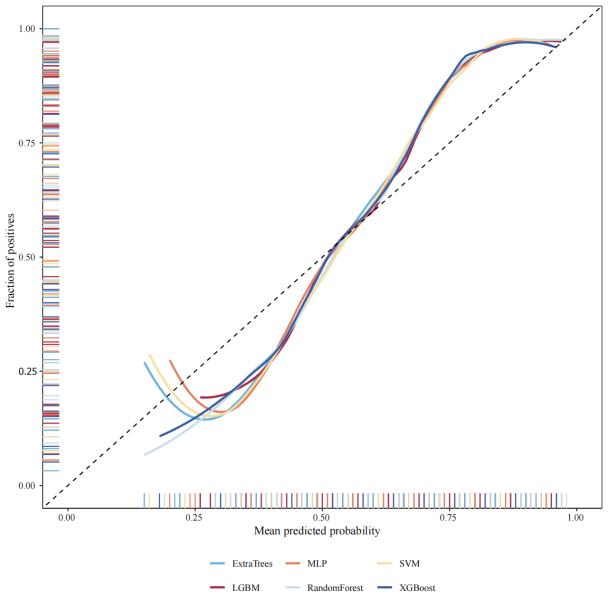

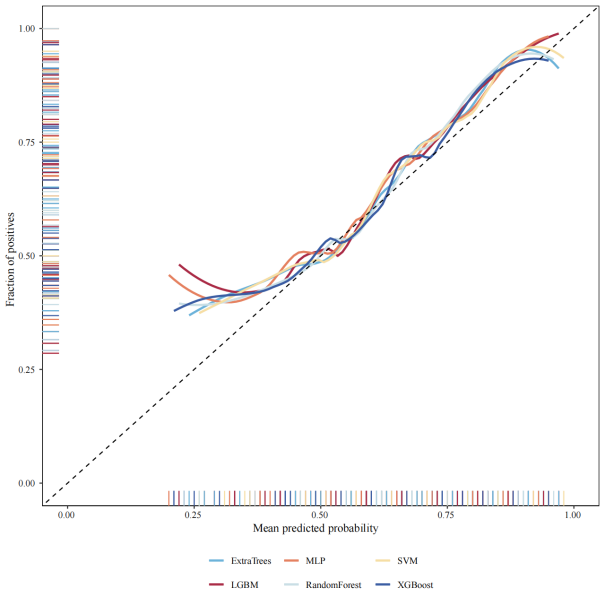


**Fig.S2** Performance comparison of ML models for pathological grade prediction in UTUC patients. (A,B) The calibration curve of the ML models in the training set and test set respectively.


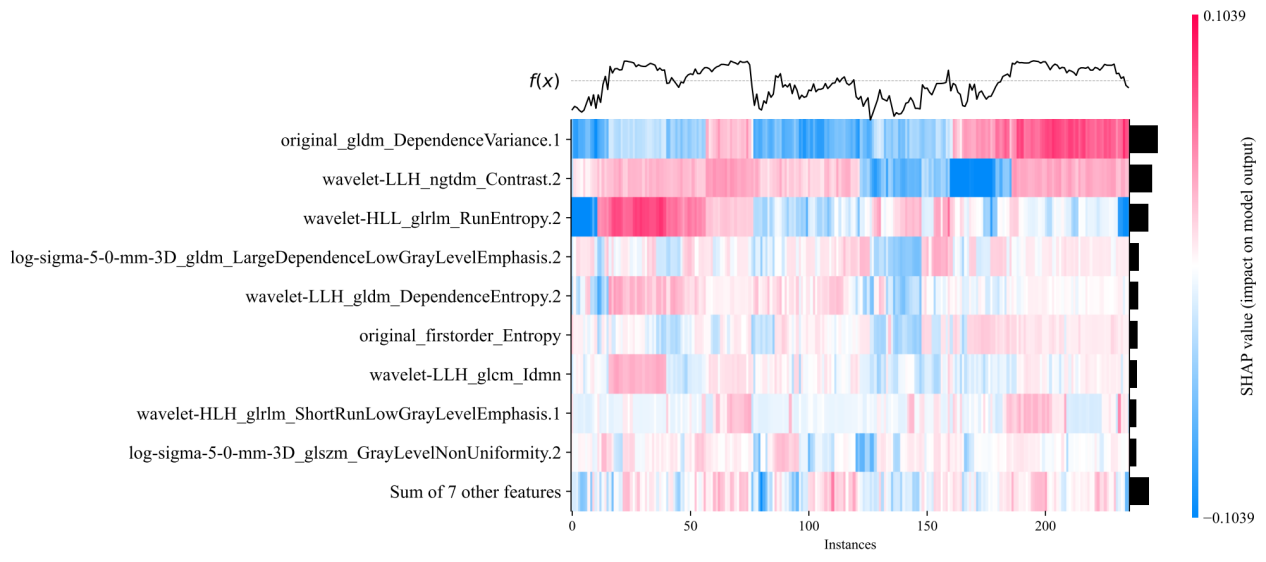


**Fig.S3** The SHAP heatmap was created to visualize feature importance and the relationships between selected radiomics features.
